# Supplementary material for: Extraterrestrial hexamethylenetetramine in meteorites—a precursor of prebiotic chemistry in the inner solar system
Source: Nat Commun. 2020 Dec 7;11:6243. doi: 10.1038/s41467-020-20038-x (PMC7721876; doi:10.1038/s41467-020-20038-x)
Supplement: Supplementary file 1 — Supplementary Information [file 41467_2020_20038_MOESM1_ESM.pdf]

Supplementary materials for:

Extraterrestrial hexamethylenetetramine in meteorites-a precursor of prebiotic chemistry in the inner solar system

Authors: Yasuhiro Oba[1*], Yoshinori Takano[2], Hiroshi Naraoka[3,4], Yoshihiro Furukawa[5], Daniel P. Glavin[6], Jason P. Dworkin[6], Shogo Tachibana[7,8]

Affiliations:

[1]Institute of Low Temperature Science (ILTS), Hokkaido University, N19W8, Kita-ku, Sapporo, Hokkaido 060-0189 Japan.

[2]Biogeochemistry Research Center (BGC), Japan Agency for Marine-Earth Science and Technology (JAMSTEC), 2-15 Natsushima, Yokosuka, Kanagawa 237-0061 Japan.

[3]Department of Earth and Planetary Sciences, Kyushu University, 744 Motooka, Nishi-ku, Fukuoka, Fukuoka 819-0395 Japan.

[4]Research Center for Planetary Trace Organic Compounds (PTOC), Kyushu University, 744 Motooka, Nishi-ku, Fukuoka, Fukuoka 819-0395 Japan.

[5]Department of Earth Science, Tohoku University, Sendai 980-8578, Japan

[6]Solar System Exploration Division, National Aeronautics and Space Administration (NASA), Goddard Space Flight Center (GSFC), Greenbelt, MD 20771, USA

[7]UTokyo Organization for Planetary and Space Science (UTOPS), University of Tokyo, 7-3-1 Hongo, Tokyo 113-0033, Japan.

[8]Institute of Space and Astronautical Science (ISAS), Japan Aerospace Exploration Agency (JAXA), 3-1-1 Yoshinodai, Sagamihara, Kanagawa 252-5210, Japan.

Contents

▪Supplementary Figures 1−12

▪Supplementary Tables 1−2

▪Supplementary Note 1

▪Supplementary References 1−25

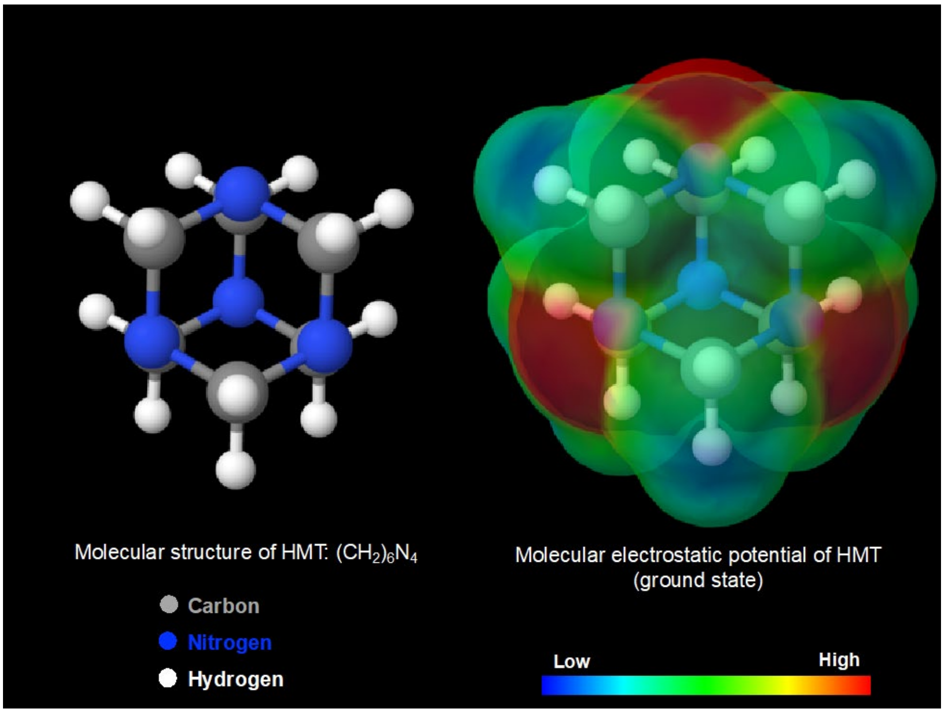

Supplementary Figure 1. Structure of hexamethylenetetramine. The symmetric chemical structure of hexamethylenetetramine and the equivalent electrostatic potential in the ground state condition.

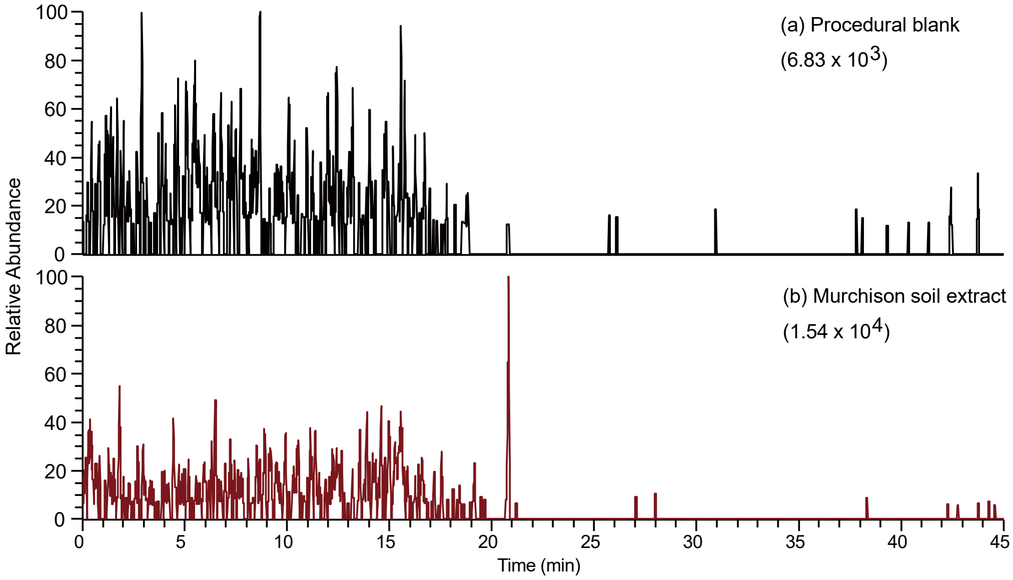

Supplementary Figure 2. Blank analyses. Mass chromatograms at the *m/z* of 141.1135 for (a) the procedural blank and (b) the Murchison soil extract at the 3-ppm window of the monoisotopic mass of the hexamethylenetetramine (HMT)-protonated ion. In panel (a), no HMT peak above the noise level exists, while in panel (b), HMT was positively identified at ~20.8 min. The HMT concentration in the Murchison soil was 2 ppb, which was ~0.2% of the Murchison meteorite extract (please see, Table 1).

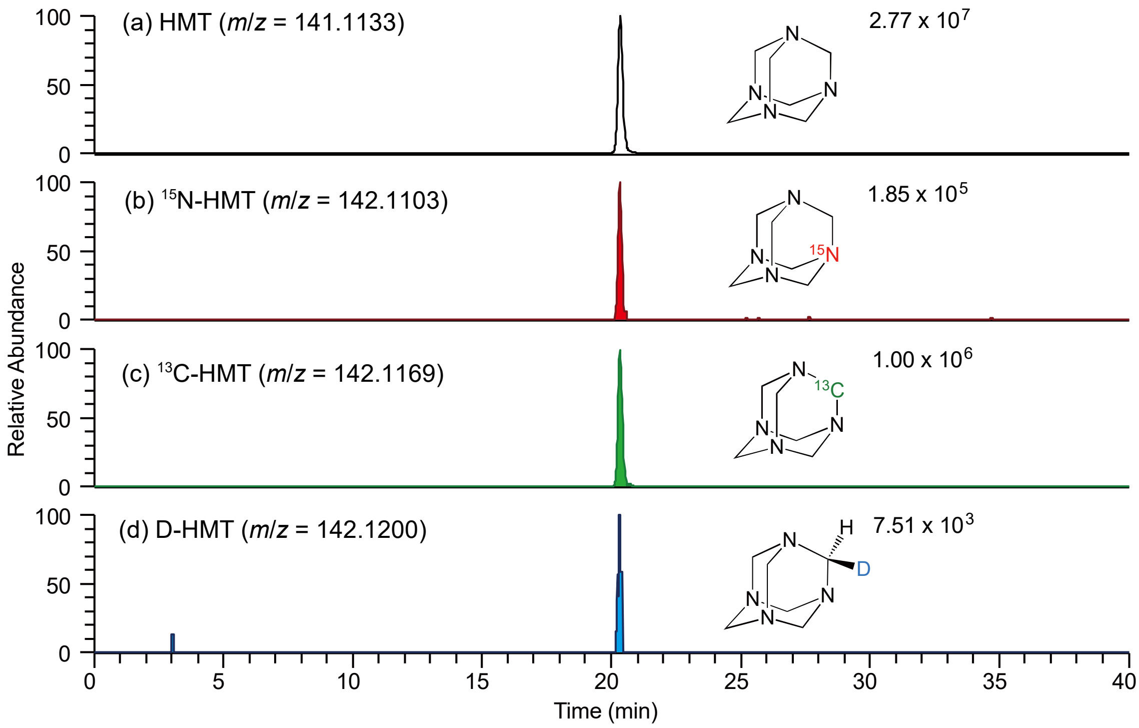

Supplementary Figure 3. Detection of hexamethylenetetramine isotopologues. Mass chromatograms extracted at the *m/z* of (a) 141.1135, (b) 142.1105, (c) 142.1168, and (d) 142.1197, which correspond to the protonated ions of hexamethylenetetramine (HMT), ^{15}N-HMT, ^{13}C-HMT, and D (^{2}H)-HMT, respectively (3-ppm window at each monoisotopic mass) in the Murchison meteorite extract. The numbers on the upper right in each panel represent the absolute scale for each chromatogram.

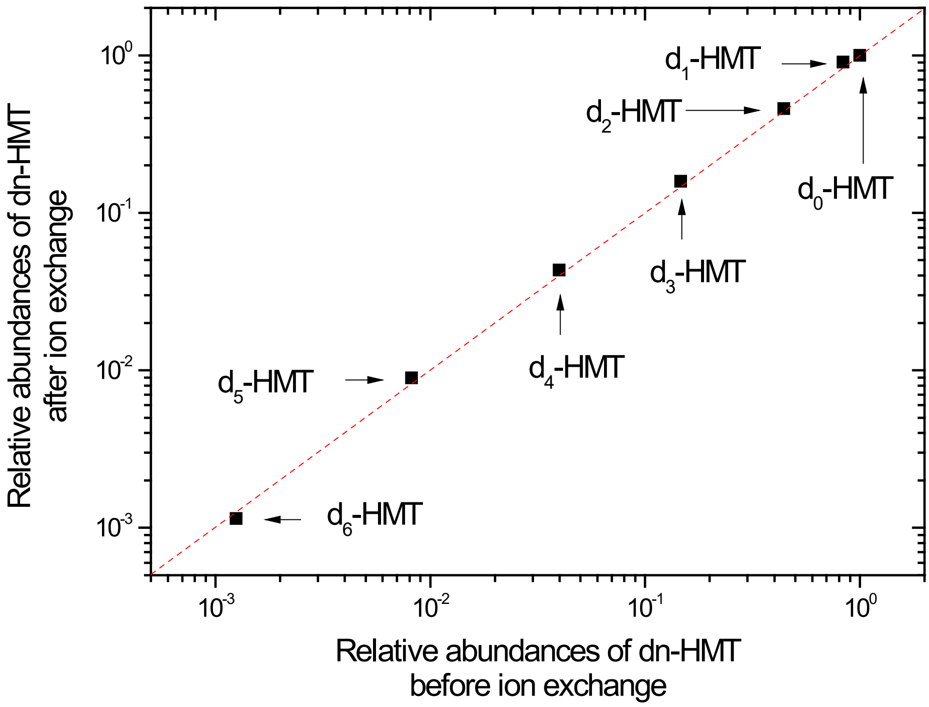

Supplementary Figure 4. Method validation. Relative abundances of deuterated hexamethylenetetramine (HMT) isotopologues (d_n-HMT, where n is the number of D atoms in an HMT isotopologue) before and after the extraction and purification procedures. The deuterated HMT isotopologues were prepared by the photolysis of ice mixtures containing a series of deuterated methanol isotopologues (Supplementary ref. 1). The sample was analysed at first without purification (concentration: X-axis). After that, the same sample was dried and processed in the same manner except the addition of the purification procedure (concentration: Y-axis). The dashed red line represents a 1:1 correlation.

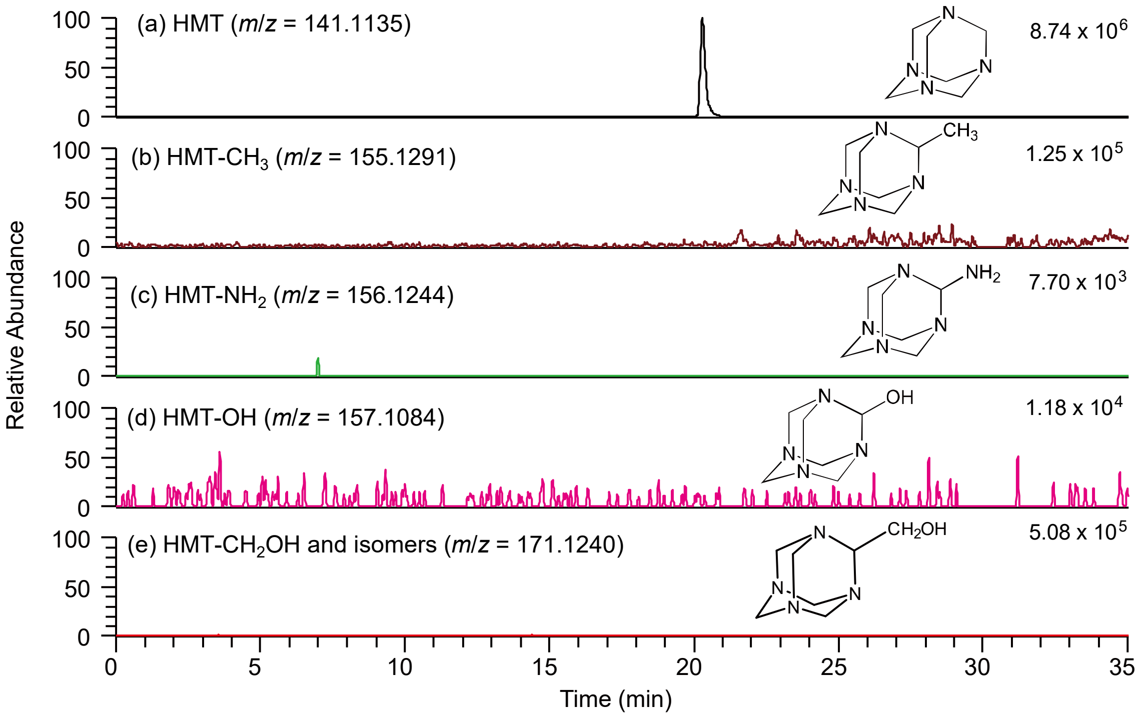

Supplementary Figure 5. Evaluation of artifact for the formation of hexamethylenetetramine derivatives. Mass chromatograms at the *m*/*z* of (a) 141.1135, (b) 155.1291, (c) 156.1244, (d) 157.1084, and (e) 171.1240, which correspond to hexamethylenetetramine (HMT), HMT-CH_3, HMT-NH_2, HMT-OH, and HMT-CH_2OH, respectively, for the HMT standard reagent (3-ppm window at each monoisotopic mass). The relative scales for each y-axis were adjusted the same as those applied in Figure 4. HMT derivatives were not identified on the mass chromatograms, indicating that the HMT-derivatives detected in the Murchison extract are not artifacts but are indigenous to the meteorite.

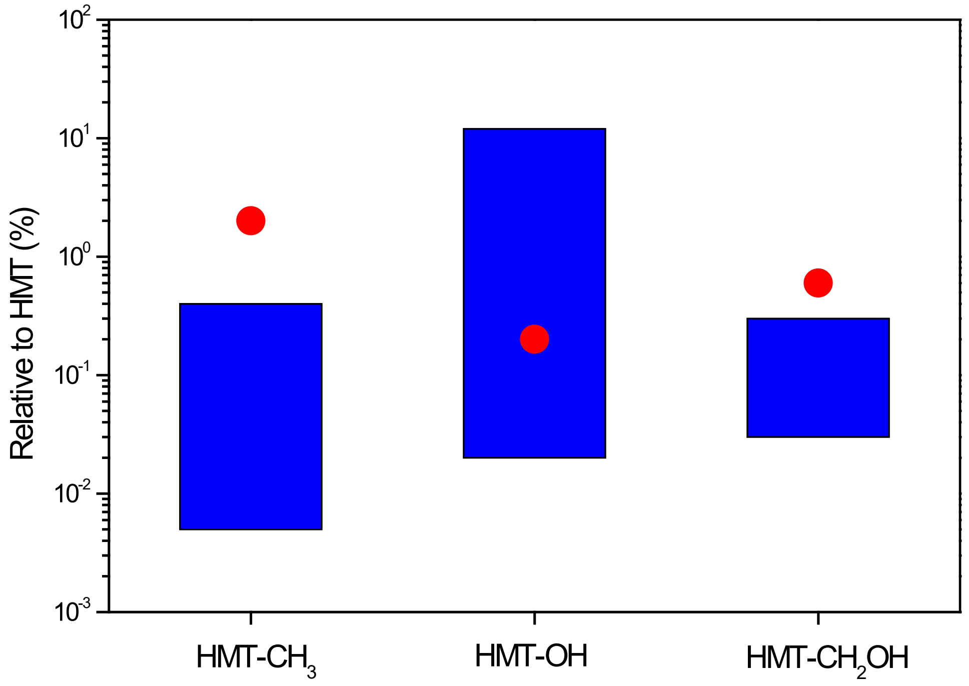

Supplementary Figure 6. Relative abundances of hexamethylenetetramine derivatives. Comparison of the relative abundance of HMT-derivatives (HMT-CH_3, HMT-OH and HMT-CH_2OH) in organic residues produced in laboratories (blue bar) (Supplementary ref. 2) and that in Murchison (red circle).

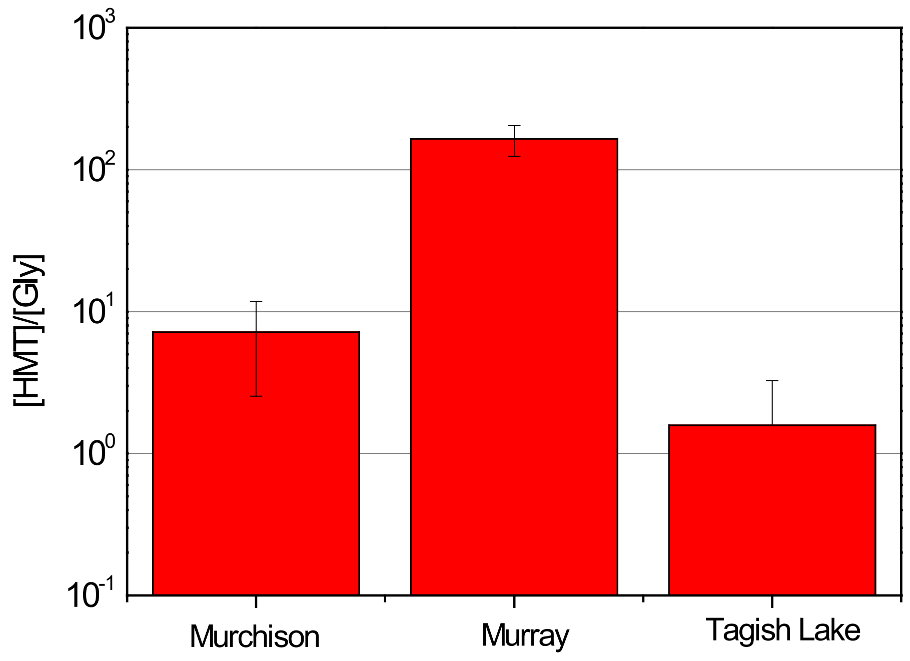

Supplementary Figure 7. Comparison with another molecule in meteorites. Variations in the relative abundance of hexamethylenetetramine with glycine ([HMT]/[Gly]) in each meteorite. The concentration of glycine was taken after Supplementary refs. 3-14. The error bars represent the standard deviation from the mean value of [HMT]/[Gly] for each meteorite. The values of [Gly] were derived from Supplementary refs. 3-11 for Murchison, Supplementary refs. 11 and 12 for Murray, and Supplementary refs. 13-15 for Tagish Lake.

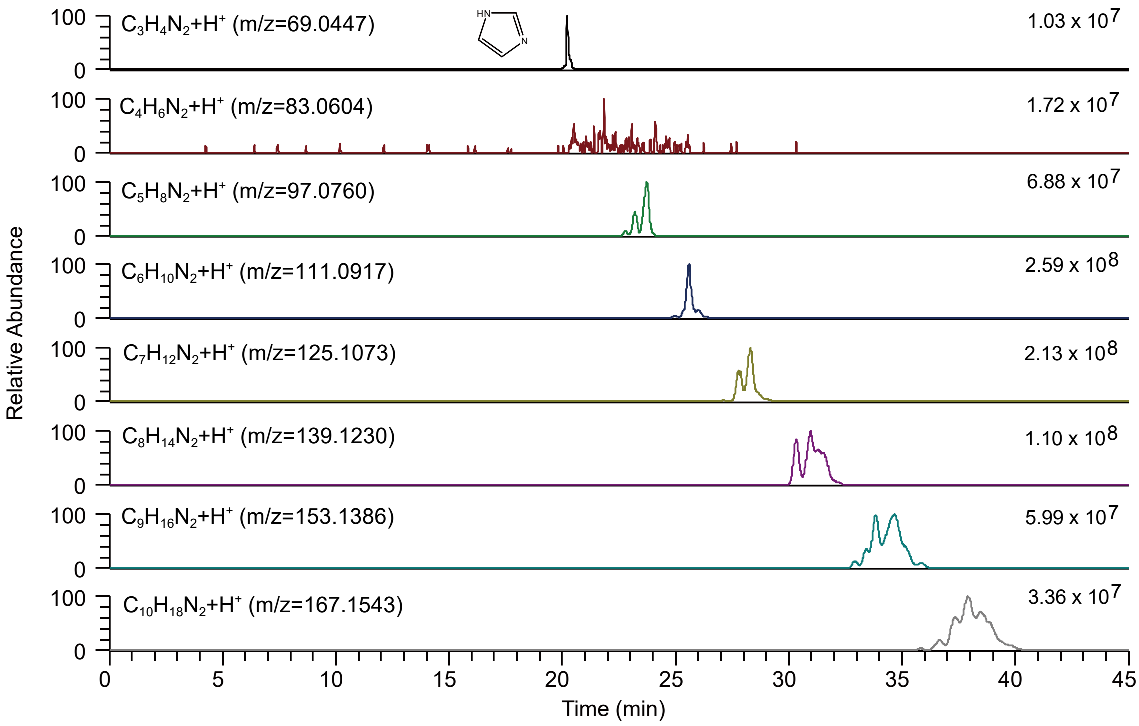

Supplementary Figure 8. Alkyl imidazoles in Murchison. Mass chromatograms at the *m/z* values corresponding to the protonated ions of alkylated imidazole series ($C_nH_{2n-2}N_2$ + H^+, n = 3 to 10) in the Murchison extract (3-ppm window at each monoisotopic mass). The values in parentheses are the theoretical masses of the alkylated imidazoles. The uppermost panel corresponds to the protonated ion of imidazole ($C_3H_4N_2$ + H^+). The values on the right side represent each absolute intensity.

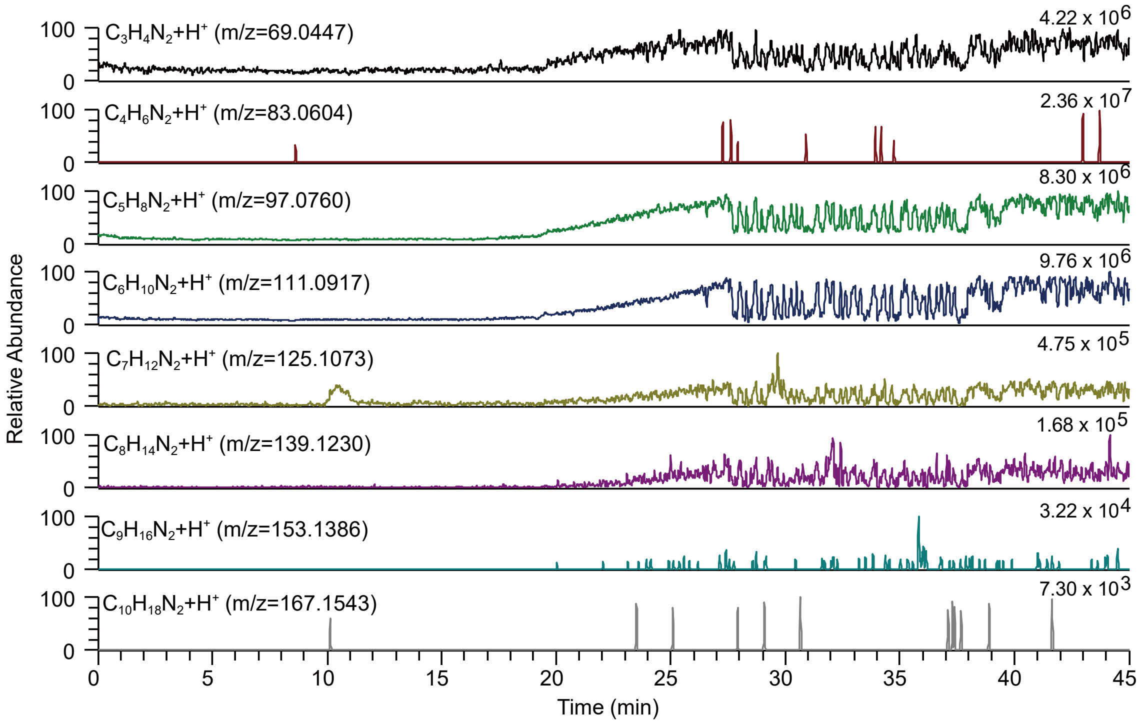

Supplementary Figure 9. Alkyl imidzoles in Tagish Lake. Mass chromatograms at the *m/z* values corresponding to the protonated ions of alkylated imidazole series ($C_nH_{2n-2}N_2 + H^+$, n = 3 to 10) in the Tagish Lake extract (3-ppm window at each monoisotopic mass). The values in parentheses are the theoretical masses of the alkylated imidazoles. The uppermost panel corresponds to the protonated ion of imidazole ($C_3H_4N_2 + H^+$). The values on the right side represent each absolute intensity.

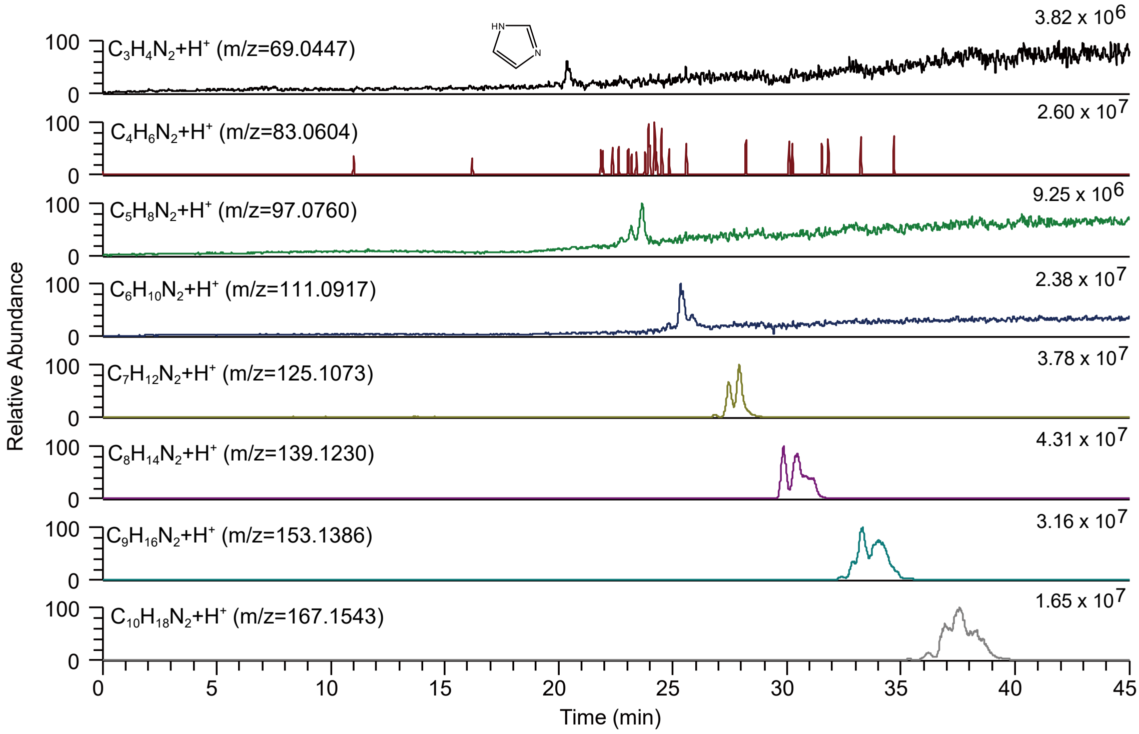

Supplementary Figure 10. Alkylimidazoles in Murray. Mass chromatograms at the *m/z* values corresponding to the protonated ions of alkylated imidazole series ($C_nH_{2n\text{-}2}N_2 + H^+$, n = 3 to 10) in the Murray extract (3-ppm window at each monoisotopic mass). The values in parentheses are the theoretical masses of the alkylated imidazoles. The uppermost panel corresponds to the protonated ion of imidazole ($C_3H_4N_2 + H^+$). The values on the right side represent each absolute intensity.

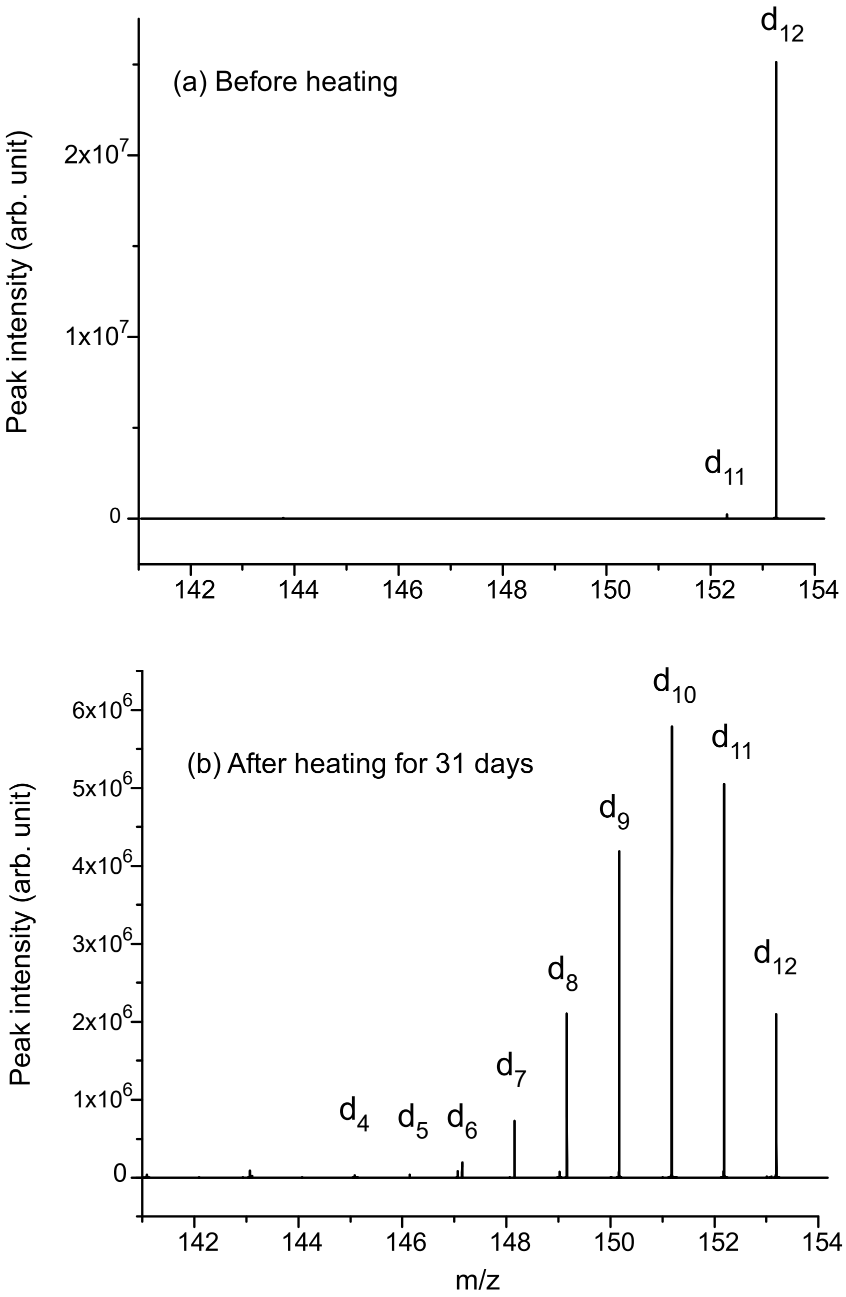

Supplementary Figure 11. Hydrogen isotopic exchange during heating with water. Variations in the mass spectra of deuterated hexamethylenetetramine (HMT-d_n) after heating with water (pH = 10) and amorphous silicates (Mg_2SiO_4) at 100 °C. A description "d_n" (n = 4–12) represents deuterated HMTs whose number of D atoms is n in HMT.

Supplementary Table 1. Summary of the retention time and the measured mass of HMT in Murchison and the standard reagent under different analytical conditions.

**Separation column**	**Retention time (min)**		**Δt (min)**	**Mass accuracy with proton**		**Δ m/z**
	HMT standard	Sample (Murchison)		Theoretical Mass $[M+H]^+$	Measured Mass $[M+H]^+$	
InertSustain PFP	20.39	20.35	0.04	141.1135	141.1133	0.0002
Hypercarb	3.79	3.78	0.01	141.1135	141.1135	0.0000
InertSustain Amide	24.86	24.78	0.08	141.1135	141.1134	0.0001

The Δt (min) value was defined as the difference in retention time (min) between the authentic HMT standard and the meteorite sample (e.g. Murchison).

Supplementary Table 2. Summary of the separation columns used in the present study.

**Separation column**	**Stationary phase**	**Separation mode**	**Specification**	**Supplier**
InertSustain PFP	Silica-based pentafluorophenyl	Reversed phase	2.1 × 250 mm, particle size of 3 µm	GL Science
Hypercarb	Graphite-based porous carbon	Reversed phase	2.1 × 150 mm, particle size of 5 µm	Thermo Fischer Scientific
InertSustain Amide	Silica-based carbamoyl	Normal phase	3.0 × 250 mm, particle size of 3 µm	GL Science

Supplementary Note 1

In addition to the examples of researches on the formation of organic molecules in asteroids as shown in the Introduction section, a number of related studies have been reported so far: the formation of IOM-like organic solids (Supplementary refs. 16–18) and sugars (Supplementary ref. 19) through polymerization of formaldehyde, the formation of amino acids (Supplementary ref. 20), alkylpyridines (Supplementary ref. 21), unidentified complex mixtures ($C_xH_yO_zN_w$) from aldehydes and ammonia (Supplementary ref. 22) under hydrothermal conditions, and the formation of nucleosides and their components from formamide with the assist of energetic protons (170 MeV) (Supplementary ref. 23). In addition, the formation of various kinds of organic molecules through the decomposition of HMT has been confirmed experimentally (Supplementary refs. 24, 25).

Supplementary References

1. Oba, Y. et al. Deuterium fractionation upon the formation of hexamethylenetetramines through photochemical reactions of interstellar ice analogs containing deuterated methanol isotopologues. *Astrophys. J.* **849**, 122 (2017).
2. Muñoz Caro, G. M. & Schutte, W. A. UV-photoprocessing of interstellar ice analogs: New infrared spectroscopic results. *Astron. Astrophys*. **412**, 121–132 (2003).
3. Glavin, D. P. et al. Extraterrestrial amino acids and L-enantiomeric excesses in the CM2 carbonaceous chondrites Aguas Zarcas and Murchison. *Meteor. Planet. Sci.* doi: 10.1111/maps.13451 (2020).
4. Engel, M. H. and Nagy, B. Distribution and enantiomeric composition of amino acids in the Murchison meteorite. *Nature* **296**, 837-840 (1982)
5. Engel, M. H., Macko, S. A. and Silfer, J. A. Carbon isotope composition of individual amino acids in the Murchison meteorite. *Nature* **348**, 47-49 (1990)
6. Cronin, J. R. and Pizzarello, S. Amino acids in meteorites. *Adv. Space Res.* **3**, 5-18 (1983)
7. Peltzer, E. T., Bada, J. L., Schlesinger, G. and Miller, S. L. The chemical conditions on the parent body of the Murchison meteorite: some conclusions based on amino, hydroxy and dicarboxylic acids. *Adv. Space Res.* **4**, 69-74 (1984)
8. Glavin, D. P. et al. Amino acid analyses of Antarctic CM2 meteorites using liquied chromatography-time of flight-mass spectrometry. *Meteor. Planet. Sci.* **41**, 889-902 (2006)
9. Botta, O., Martins, Z. and Ehrenfreund, P. Amino acids in Antarctic CM1 meteorites and their relationship to other carbonaceous chondrites. *Meteor. Planet.*

Sci. **42**, 81-92 (2007)

10. Burton, A. S. et al. The amino acid composition of the Sutter's Mill CM2 carbonaceous chondrite. *Meteor. Planet. Sci.* **49**, 2074-2086 (2014)
11. Ehrenfreund, P. et al. Extraterrestrial amino acids in Orgueil and Ivuna: Tracing the parent body of CI type carbonaceous chondrites. *Proc. Nat. Acad. Sci. USA* **98**, 2138–2141 (2001).
12. Cronin, J. R. and Moore, C. B. Amino acid analyses of the Murchison, Murray, and Allende carbonaceous chondrites, *Science*, **172**, 1327-1329 (1971)
13. Glavin, D. P. et al. Unusual nonterrestrial L-proteinogenic amino acid excesses in the Tagish Lake meteorite. *Meteor. Planet. Sci.* **47**, 1347–1364 (2012).
14. Hilts, R. W., Herd, C. D. K., Simkus, D. N. and Slater, G. F. Soluble organic compounds in the Tagish Lake meteorite. *Meteor. Planet. Sci.* **49**, 526-549 (2014)
15. Kminek, G., Botta, O., Glavin, D. & Bada, J. Amino acids in the Tagish Lake meteorite. *Meteor. Planet. Sci.* **37**, 697–701 (2002).
16. Cody, G. D. et al. Establishing a molecular relationship between chondritic and cometary organic solid. *Proc. Natl. Acad. Sci. USA*, **108**, 19171–19176 (2011).
17. Kebukawa, Y., Kilcoyne, A. L. D. and Cody, G. D. Exploring the potential formation of organic solids in chondrites and comets through polymerization of interstellar formaldehyde. *Astrophys. J.*, **771**, 19 (2013).
18. Kebukawa, Y. and Cody, G. D. A kinetic study of the formation of organic solids from formaldehyde: Implications for the origin of extraterrestrial organic solids in primitive Soar System objects. *Icarus*, **248**, 412–423 (2015).
19. Furukawa, Y. et al. Extraterrestrial ribose and other sugars in primitive meteorites. *Proc. Natl. Acad. Sci. USA* **116**, 24440–24445 (2019).

20. Koga, T. & Naraoka, H. A new family of extraterrestrial amino acids in the Murchison meteorite. *Sci. Rep.* **7**, 636 (2017).
21. Yamashita, Y. and Naraoka, H. Two homologous series of alkylpyridines in the Murchison meteorite. *Geochem. J.*, **48**, 519–525 (2014).
22. Isono, Y. et al. Bulk chemical characteristics of soluble polar organic molecules formed through condensation of formaldehyde: Comparison with soluble organic molecules in Murchison meteorite. *Geochem. J.*, **53**, 41–51 (2019).
23. Saladino, R. et al. Meteorite-catalyzed syntheses of nucleosides and of other prebiotic compounds from formamide under proton irradiation. *Proc. Natl. Acad. Sci. USA* **112**, E2746–E2755 (2015).
24. Vinogradoff, V., Bernard, S., Le Guillou, C. & Remusat, L. Evolution of interstellar organic compounds under asteroidal hydrothermal conditions. *Icarus* **305**, 358–370 (2018).
25. Vinogradoff, V. et al. Influence of phyllosilicates on the hydrothermal alteration of organic matter in asteroids: Experimental perspectives. *Geochim. Cosmochim. Acta*, **269**, 150–166 (2020).
